# Bottom-up device fabrication *via* the seeded growth of polymer-based nanowires†
†Electronic supplementary information (ESI) available: Experimental details of the polymer preparation, growth of micelles, characterisation and data analysis (cyclic voltammetry, AFM and electrical measurements). See DOI: 10.1039/d0sc02011g


**DOI:** 10.1039/d0sc02011g

**Published:** 2020-06-01

**Authors:** Osama El-Zubir, Emily L. Kynaston, Jessica Gwyther, Ali Nazemi, Oliver E. C. Gould, George R. Whittell, Benjamin. R. Horrocks, Ian Manners, Andrew Houlton

**Affiliations:** a Chemical Nanoscience Labs , School of Natural and Environmental Sciences , Newcastle University , Newcastle upon Tyne NE1 7RU , UK . Email: andrew.houlton@ncl.ac.uk; b School of Chemistry , University of Bristol , Cantock's Close , Bristol BS8 1TS , UK; c Department of Chemistry , University of Victoria , Victoria , V8W 3V6 , British Columbia , Canada . Email: imanners@uvic.ca

## Abstract

The bottom-up assembly of nanoelectronic devices from molecular building blocks is a target of widespread interest. Herein we demonstrate an *in situ* seeded growth approach to produce a nanowire-based electrical device. This exploits the chemisorption of block terpolymer-based seed fibres with a thiophene-functionalised corona onto metal electrodes as the initial step. We then use these surface-bound seeds to initiate the growth of well-defined one-dimensional fibre-like micelles *via* the seeded growth method known as “Living crystallisation-driven self-assembly’’ and demonstrate that they are capable of spanning an interelectrode gap. Finally, a chemical oxidation step was used to transform the nanofibres into nanowires to generate a two-terminal device. This seeded growth approach of growing well-defined circuit elements provides a useful new design tool for bottom-up device fabrication.

## Abstract 2

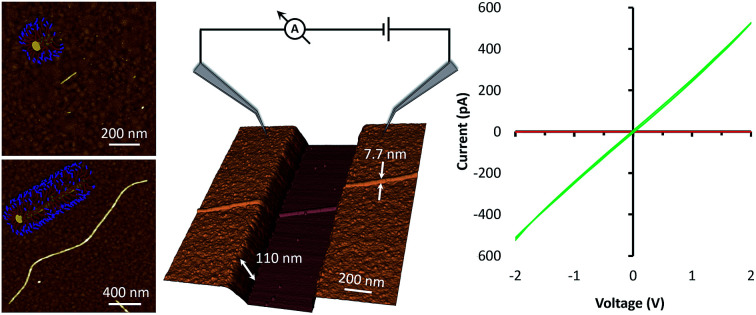
Living crystallisation-driven self-assembly facilitates the bottom-up assembly of electronic devices.

## Introduction

The bottom-up fabrication of nanoelectronic devices was conceptualised decades ago^1^,^2^ but remains as an important challenge of intense interest. Overwhelmingly, in examples to date, preformed components, ranging from small single molecules to anisotropic nanostructures *e.g.* carbon nanotubes, are directed towards the inter-electrode region to complete an electrical circuit. Simple circuits have thus been assembled using a variety of approaches for component organisation such as flow,^3^–^5^ electrophoresis,^6^ dipping,^7^ hydrogen bonding/molecular recognition (DNA),^2^ chemisorption in a nanogap,^8^ and dip-pen lithography.^9^

An alternative, much less explored, approach is to extend the self-assembly stages to the components themselves and for these to be grown *in situ*. 10–13 This could, in principle, allow components to adapt to changes in device configuration, such as electrode spacing, number *etc.* and enable more complex architectures to be constructed from simple molecular building blocks. However, previous efforts have relied on simple electropolymerisation with the resulting lack of precise control in the morphology of the resulting interconnects leading to poor definition of morphology and size.^10^–^12^

Living crystallisation-driven self-assembly (CDSA) is a well-established seeded growth approach to uniform 1D^14^–^20^ and 2D^21^–^28^ nanostructures using amphiphilic polymeric or molecular building blocks. The living CDSA process resembles a living covalent polymerisation except that it occurs on a longer lengthscale and involves an epitaxial growth mechanism.^14^,^29^ Ultrasonication of fibre-like micelles with a crystalline core leads to fracture by Gaussian scission to give shorter micelles which function as seed initiators for fibre-like micelle growth by living CDSA.^29^ The addition of further block terpolymer in a molecularly dispersed form (unimers) leads to epitaxial growth at the seed termini to yield uniform fibre-like micelles with length dependent on the mass ratio of the added unimer to the pre-existing seeds. Epitaxial growth of fibre-like block terpolymer micelles has been achieved from the edges of platelet micelles, from the surface of thin films of homopolymer^30^ and from seed micelles localised on silica, colloidosome, and silicon surfaces.^31^–^34^

In a previous study, CDSA has been used to assemble well-defined templates for the preparation of polyaniline nanowires with controlled lengths in solution-phase.^35^ Here we exploit this CDSA behaviour to demonstrate an *in situ* approach to construct a simple electrical circuit using redox-active fibre-like block terpolymer micelles as building blocks. Specifically, we demonstrate that block terpolymer seeds from Gaussian scission of micelles can be designed to chemisorb onto electrode surfaces and initiate the controlled interfacial growth of micelles of well-defined length. Further, these fibre-like micelles are capable of spanning an interelectrode gap and may then be switched to a more electrically conducting “wire” form by chemical oxidation.

## Results and discussion

The procedure used to prepare the triblock terpolymer PFDMS_44_-*b*-PDMS_250_-*b*-P3OT_17_ and to form seed micelles from this material is described in the ESI.†

### Chemisorption and activity of surface-bound fibre-like micelle seeds

**Fig. 1:**
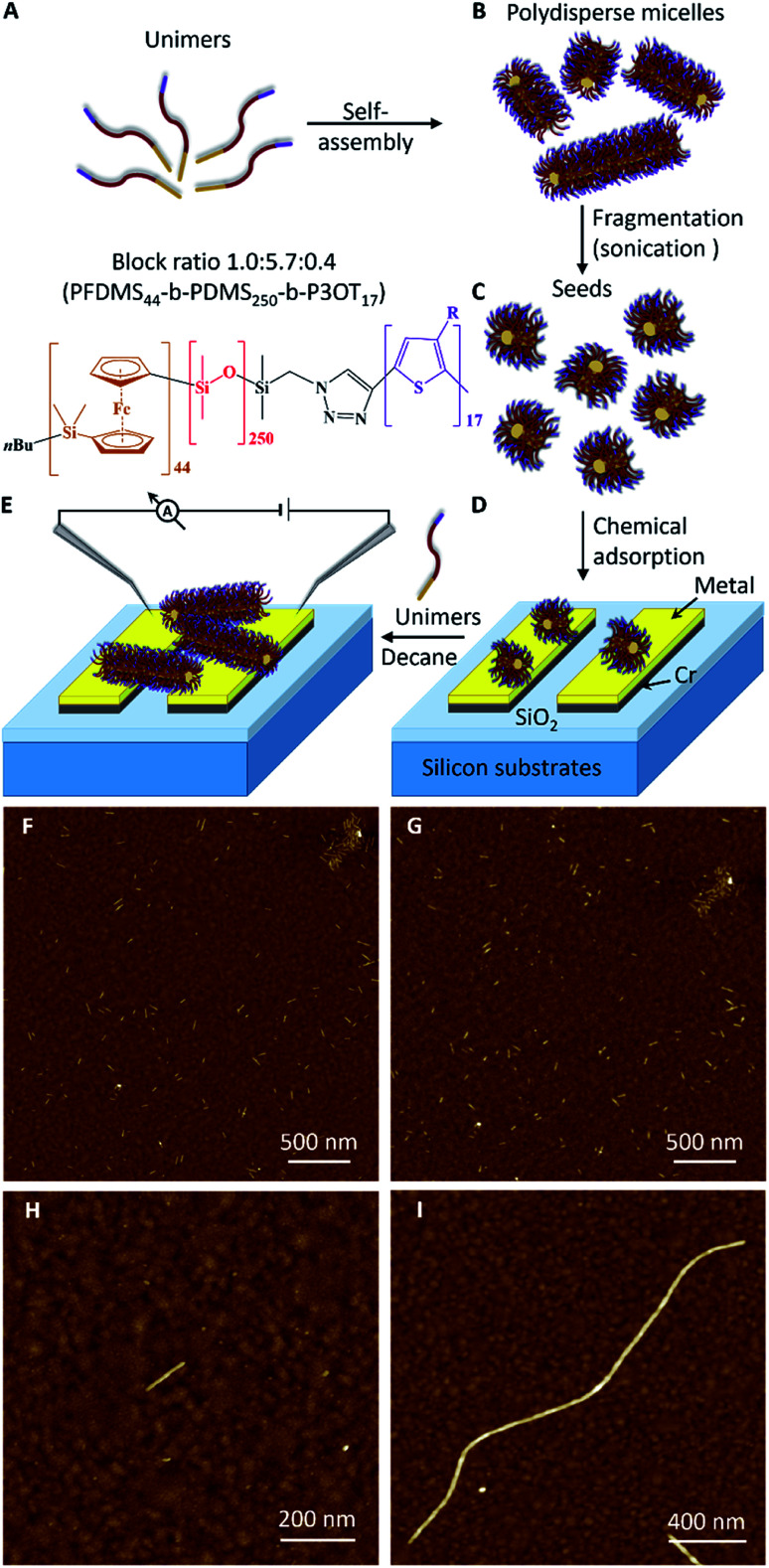
Growth of fibre-like micelles *via* the living crystallisation-driven self-assembly of PFDMS block terpolymers. (A) Schematic representation of the formation of cylindrical micelles. The chemical structure of unimers are shown in orange (PFDMS), red (PDMS) and purple (P3OT). (B) Self-assembly of PFDMS_44_-*b*-PDMS_250_-*b*-P3OT_17_ unimers to form polydisperse micelles. (C) Fragmentation of the micelles using sonication to yield seeds. (D) Chemical adsorption of seeds on a metal (Au or Pt) electrode surface. (E) Growth of a fibre-like micelle from adsorbed seeds to cross the inter-electrode gap of an electrical device. (F and G) AFM images of adsorbed seeds on gold surface after immersion for 2 h in a 0.5 μg ml^–1^ solution in decane. The surfaces were then washed by immersion for (F) 2 min and (G) 24 h in pure decane. (H) Image of a seed adsorbed on a gold substrate from a 0.005 μg ml^–1^ solution of seeds immersed for 2 h. (I) Image of a micelle grown from an adsorbed seed on gold by immersion in 4 μg ml^–1^ solution of unimers for 18 h.

Fig. 1 outlines the scheme for device assembly based on this three-step seeded-growth approach. Here, in this proof of concept work, we use a triblock terpolymer poly (ferrocenyldimethylsilane)-*block*-poly(dimethylsiloxane)-*block*-poly(3-octylthiophene) (PFDMS_44_-*b*-PDMS_250_-*b*-P3OT_17_). The design criteria for this material were to use the well-characterised, crystallisable PFDMS^36^ core-forming block for living CDSA and an electroactive, π-conjugated polymer, poly(3-octylthiophene) (∼17-mer), the peripheral coronal segment with a central PDMS corona-forming block inbetween. The P3OT block was incorporated into the polymer to play two roles in the fibre-like micelle structure. First, the conjugated thiophene block can be switched to an electronically conducting form after doping.^37^,^38^ Second, thiophene residues should facilitate attachment of the seeds to the gold surface by spontaneous chemisorption.^39^,^40^

In the initial stage towards device assembly we sought to confirm chemisorption of seeds at metal surfaces. As a simple proof, Au-on-Si substrates were immersed in a solution of PFS-*b*-PDMS-*b*-P3OT seeds in decane. Cyclic voltammetry of these substrates exhibited, after extensive washing, the expected redox behaviour for the ferrocenyl/thiophene-containing polymer (ESI Fig. S4†). The chemisorption was further confirmed by AFM which also allowed the stability of the attachment to be monitored. Scanning the same area of the modified substrate (Fig. 1F, G and S5A–E†) showed that no seeds were removed from the gold surface after immersion in decane for 2, 4, 6, and 24 hours (see details in the ESI†).

AFM imaging (Fig. 1H) shows a single adsorbed seed on a gold surface that is about 120 nm in length. Such modified surfaces act to seed the growth of fibre-like micelles and confirm that the ends of the seeds remain active even after the chemisorption on the gold surface. Fig. 1I shows a fibre-like micelle self-assembled on the gold surface from an adsorbed seed by immersion in a solution of unimers. The fibre-like micelle is well defined with uniform diameter of 7.7 nm (as expected from simple models) and extends 2.4 μm in length. Such a well-defined size and morphology of the fibre-like micelle is in marked contrast to the (electro)chemically grown polymer systems.^10^–^12^ The AFM data reveal the living nature of the CDSA process^29^ by showing clear evidence for the increase in the seed length. In contrast, a control sample (Fig. S5F†) of a pre-cleaned gold substrate immersed for 18 h in a decane solution of unimers showed no micelles, confirming that the living CDSA process by self-initiation is inefficient over this timeframe. Seeding the surface is much more efficient than self-nucleation of fibre-like micelles in a similar manner to the growth of the fibre-like micelles in homogeneous solution.^30^

### Control of seed density and fibre-like micelle length on gold surfaces

**Fig. 2:**
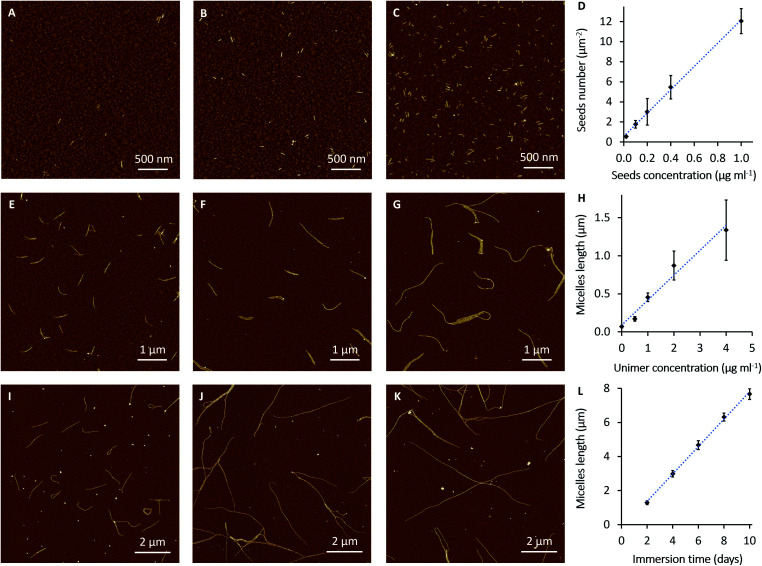
AFM images of seeds and fibre-like micelles of PFDMS_44_-*b*-PDMS_250_-*b*-P3OT_17_ on gold surfaces. The gold coated silicon slides were immersed for 2 h in seeds solution (A) 0.02 μg ml^–1^, (B) 0.1 μg ml^–1^ and (C) 1 μg ml^–1^. (D) Variation in number of seeds as function of seeds solution concentration. (E–G) The micelles were grown from 0.02 μg ml^–1^ immobilised seeds on Au surfaces by immersion for 1 day in unimer solutions with concentrations (E) 1, (F) 2 and (G) 4 μg ml^–1^. (H) Variation in length of micelles after 1 day as a function of unimer solution concentration. (I–K) Slow growth of the micelles from 0.005 μg ml^–1^ seeds on gold surfaces and immersion in 0.5 μg ml^–1^ of unimers solution for (I) 2, (J) 6 and (K) 10 days. (L) Variation in length of micelles as function of immersion time.

Next, we set out to establish details of the experimental conditions required for optimising the seed density on the electrode surface and for control of micelle length in an effort to provide parameters for device construction. In these experiments, seeds at different concentrations (0.02, 0.1, 0.2, 0.4 and 1 μg ml^–1^) were diluted in 1 ml of decane and allowed to interact with the gold surfaces (Au-on-Si; 8 × 8 mm). Immobilisation was judged to occur in appreciable numbers after around 7 min. However, all the samples have been immersed for 2 h in order to increase the loading of adsorbed seeds. Fig. 2A–C shows AFM images of PFDMS-*b*-PDMS-*b*-P3OT seeds immobilised on gold substrates. The number of the adsorbed seeds on the surface increased in proportion to the concentration of seeds in solution, as expected (Fig. 2D). Importantly, this makes it possible to grow a desired number of micelles per micrometer by adjusting the concentration of the seed solution. From this analysis, solutions of 0.02 μg ml^–1^ or less were considered to produce the most suitable density of seeds on the surface for fibre-like micelle growth. This concentration range allows sufficient space for the micelles to grow on the surface with minimum crossing.

Growth of fibres with well-controlled length from the adsorbed seeds is an important factor for fabrication of nanowire devices. Controlling the length of the fibre-like micelles by variation of the unimer-to-seed ratio in a solution was previously demonstrated;^29^,^41^ here we investigate the kinetics of the process on solid surfaces. Seeds were adsorbed onto gold-coated substrates from solutions by immersion for 2 h. These substrates were then washed with decane and immersed in different concentrations of PFDMS-*b*-PDMS-*b*-P3OT unimer solutions overnight (18 h). The concentrations of the unimer solutions were 0.5, 1, 2 and 4 μg ml^–1^. AFM images in Fig. 2E–G show that the degree of control of the growth process depends on the concentration of the unimer solution. The data analysis in Fig. 2H shows the variation in fibre-like micelle length as a function of unimer solution concentration. The average contour lengths of the micelles were determined by tracing the length of 50 fibre-like micelles from at least 6 AFM images on randomly selected areas of each sample. It is clear from these data that the micelles grow to greater lengths as the unimer concentration is increased. However, it was found that micelles assembled from unimer solutions of 4 μg ml^–1^ and higher, start to aggregate and form networks on the surface, (Fig. S6†). It can also be judged from AFM images (*e.g.*Fig. 2G) and the error bars in Fig. 2H that fibre-like micelles grow with more variability of length at higher concentrations of unimer solution.

### Kinetic analysis of fibre-like micelle length distributions on gold surfaces

Owing to the limitations on control of the fibre-like micelle length using the concentration of the unimer solutions, the effect of immersion time was studied in order to identify conditions for controlled growth of long monodisperse micelles at surfaces. Our experimental results indicate that living CDSA to form PFDMS_44_-*b*-PDMS_250_-*b*-P3OT_17_ fibre-like micelles can continue for weeks. The AFM images in Fig. S7† were obtained by adsorption of seeds from 0.02 μg ml^–1^ seed solutions and growth of the fibre-like micelles by immersion in 2 μg ml^–1^ unimer solutions in decane for one day and 20 days, respectively. In the sample immersed for one day (Fig. S7A†) the micelles have grown to less than 1 μm. By comparison after 20 days the micelles have grown over 10 microns in length and formed a dense network (Fig. S7B†).

In Fig. 2I–K, the fibre-like micelles were self-assembled from surfaces with low numbers of seeds (adsorbed from a 0.005 μg ml^–1^ seeds solution) in order to form a less dense network of long fibres which are amenable to quantitative analysis. The fibre-like micelles were left to grow in 0.5 μg ml^–1^ unimer solution in decane for different immersion times (2, 4, 6, 8 and 10 days). The relationship (Fig. 2L) between the immersion time and the fibre-like micelle length shows a high degree of control over the self-assembly process. In this relationship, the small error bars indicate a more uniform rate of the fibre-like micelle growth compared to Fig. 2H. The AFM images and the data analysis of this slow growth of the fibre-like micelles show that these may be grown precisely to a desired length by controlling the immersion time of adsorbed seeds in the unimer solution at an appropriate concentration.

The kinetic data in Fig. 2I–K can be analysed further by considering the growth process as an example of chain growth polymerisation. Such a mechanism, in which growth occurs solely by addition of the unimers to the end of the fibre-like micelle and for which there is no termination, yields a probability mass distribution of Poisson form as in eqn (1).1
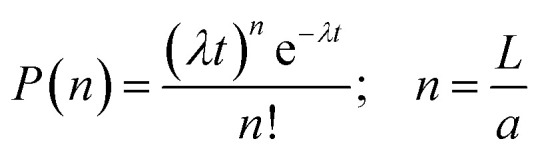



The parameter *λ* is the mean rate of growth of micelles and is a pseudo-first order rate constant proportional to the unimer concentration (μg ml^–1^) as shown in Fig. 2E–H. We estimate a value of *aλ* = 8.0 ± 0.20 × 10^–1^ μm day^–1^ from the data of Fig. 2L by linear regression.

**Fig. 3:**
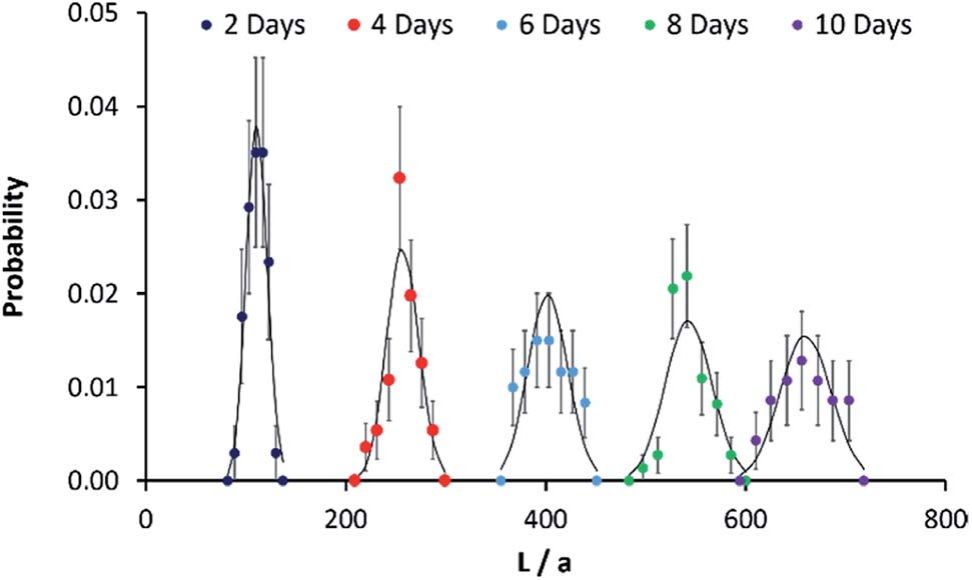
Fibre-like micelle length distributions against reaction time. Probability mass functions for the length of micelles grown on gold-coated silicon slides in Fig. 2L. The solid black line shows the Poisson distribution at each time point and the *x*-axis is given in terms of a normalised length described in the text. The micelles were grown from 0.005 μg ml^–1^ seeds on gold surfaces and immersion in 0.5 μg ml^–1^ of unimers solution for the times stated on the legend. The probabilities were estimated using AFM to measure the length of 50 separate micelles (30 in the case of the 10 day time point); standard deviations on each data point were obtained by applying counting statistics.

In order to compare the experimental data on length distributions to eqn (1), we need to estimate the value of the parameter *a* in order to convert the measured lengths to integer numbers of fundamental units. The correct value of *a* is not necessarily the size of a single unimer, because growth may occur in spurts if a particular repeat unit is more stable than average. However, *a* may be estimated from the data by linear regression of the variance of the lengths on the mean lengths as a function of time (details in the ESI Fig. S8†). We obtain a value of *a* = 11.6 ± 0.3 nm. This is very close to the value of 11.6 ± 1.0 nm for PI_550_-*b*-PFS_50_ fibre-like micelles which was derived by applying the analysis based on eqn (1) to the solution phase data of previous work^29^ and of a similar order to the value of 16.1 ± 3.9 nm for PFS-*b*-(PEO-*g*-TEG).^41^ Once the values of *a* and *λ* are determined, there are no other free parameters and Fig. 3 compares the Poisson distribution to the experimental data. The agreement of eqn (1) with the data supports a living chain growth mechanism which explains the origin of the narrow length distributions in these structures. In comparison with a step growth process, in which fibre-like micelles may add to each other, the length distribution in chain growth is much narrower. In this example it is worth noting that the seeds and fibre-like micelles are anchored on a surface and are immobile on the experiment timescale.

To test the generality of this method we also confirmed that surface-grown fibre-like micelles could be selectively functionalised, as is known for solution-based assembly.^14^,^42^ It was demonstrated that fibre-like micelles could be grown from surface-bound seeds from different unimer solutions (see details in the ESI Fig. S9†). In this way parts of the fibre-like micelles could be selectively functionalised with different functional groups.

### Fibre-like micelle growth across a gap

**Fig. 4:**
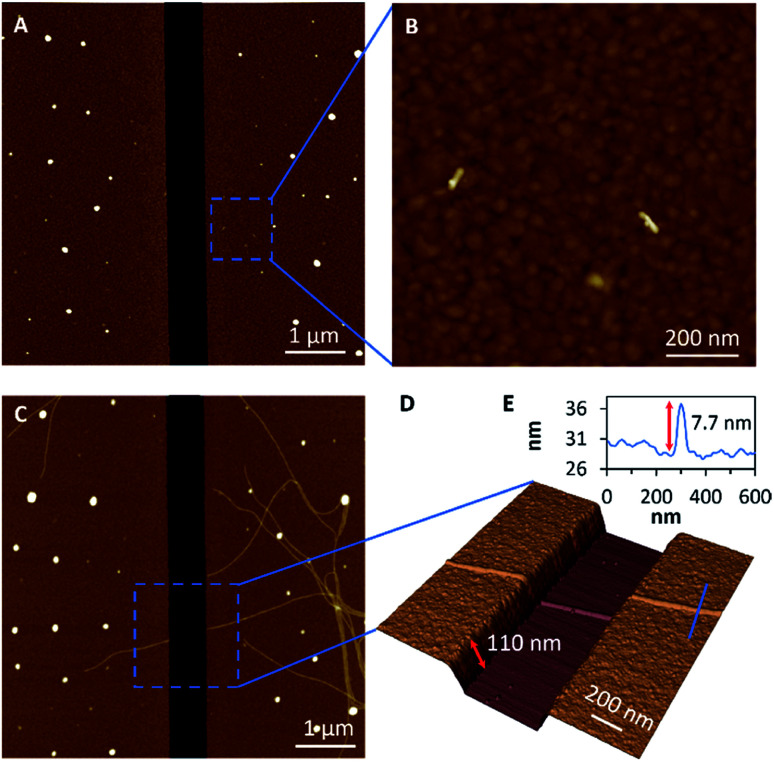
Growth of fibre-like micelles across an electrode gap. (A) A large area scan and (B) a magnified area of AFM height images of seeds were adsorbed on gold electrodes from 0.005 μg ml^–1^ solution of seeds. (C) AFM large-scan height image and (D) a 3D magnified area of the AFM height image of micelles were grown up from the adsorbed seeds on gold electrodes by immersion in 1 μg ml^–1^ solution of unimers for 5 days. (E) The associated cross-section along the blue line in (D) shows the height of the micelle.

Having established appropriate conditions for the chemisorption of seeds and subsequent fibre-like micelle growth at gold surfaces, we next performed similar experiments at gold films which contain a 600 nm gap. The terpolymer seeds were adsorbed on the clean gold surface by immersion in a solution (0.005 μg ml^–1^) in decane for 1 h to provide small numbers of seeds on the surfaces, as shown by AFM in Fig. 4A and B. Next, fibre-like micelles were self-assembled on the gold surface by immersion in a solution of unimer (1 μg ml^–1^) in decane for 5 days. The micelles grow to about 4 μm in length and readily span the gap of 600 nm (Fig. 4C). The three-dimensional AFM image in Fig. 4D shows detail of the fibre-like micelle structure crossing the 600 nm gap. These data show the remarkable tolerance of the micelle formation by the living CDSA process as it navigates height changes of over 100 nm between the underlying silicon substrate and the deposited gold. This demonstration of conformal tracking of a thin (7 nm) fibre-micelle across the device is a useful feature of the self-assembly process and highlights excellent tolerance of changes in both substrate height and material composition.

### Device fabrication and electrical measurements

**Fig. 5:**
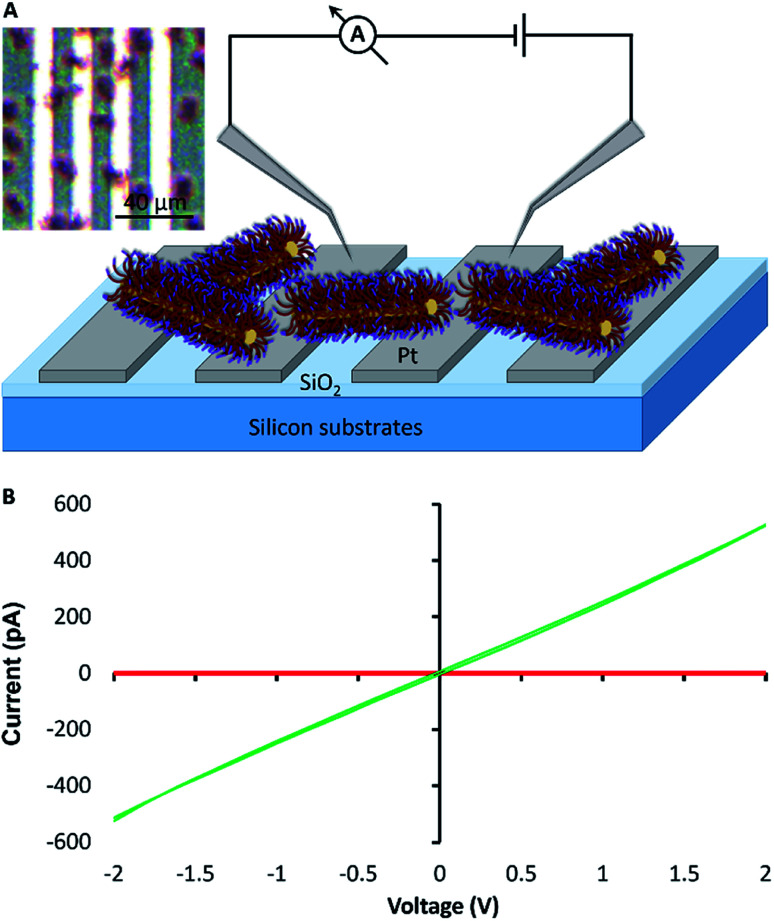
Fabrication of a simple mesoscopic electrical device. (A) Schematic representation of a simple electrical circuit constructed from bundlesv of fibre-like terpolymer micelles grown on platinum microband electrodes. (B) *I*–*V* measurements of the fibre-like micelles before (red) and after (green) doping with NOBF_4_. Inset, an optical image of bundles of fibre-like micelles grown from adsorbed seeds on a platinum microband electrode (MBE). Bundles of fibre-like micelles were grown from a high concentration of seeds (0.1 μg ml^–1^, 10 min) and unimer (10 μl ml^–1^, 7 days); they appear as red spots on the platinum MBE in the optical micrograph.

For electrical measurements, and to further test the length-scale of the assembly process, bundles of fibre-like block terpolymer micelles were grown on patterned platinum microband electrodes (MBE) with a 10 μm inter-electrode gap (Fig. 5A and S10†). Current–voltage curves collected for the as-prepared fibre-like micelles showed negligible current was passed when a voltage in the ±2 V range was applied to the device. However, a readily measurable current could be passed after doping the bundles of fibre-like micelles with the oxidising agent nitrosonium tetrafluoroborate (NOBF_4_). *I*–*V* curves collected after this treatment (Fig. 5B) show an expected increase in conduction consistent with oxidative doping of the polythiophene.^43^ Control experiments where NOBF_4_ was applied to MBEs in the absence of fibre-like micelles showed negligible background currents (Fig. S11†).

## Conclusions

In this study, we have established a detailed protocol for the growth of well-defined polymer-based nanowires based on PFDMS-*b*-PDMS-*b*-P3OT fibre-like micelles across electrode gaps to fabricate simple electrical circuits. This was demonstrated over two quite different length scales and shows that the growth process is conformal over considerable changes in substrate height and compatible with commercially available microelectrodes.

The fibre-like micelles were grown from chemisorbed seeds on the gold surfaces *via* living CDSA using PFDMS triblock terpolymer. AFM, supported by cyclic voltammetry, demonstrates the spontaneous adsorption of the seeds onto metal electrodes and their suitability for growing fibre-like micelle structures with a range of functionality at electrode devices. The seed ends can be kept active for device construction and growth by storage in THF at room temperature for up to 6 months.

A comprehensive study of the self-assembly process for the PFDMS_44_-*b*-PDMS_250_-*b*-P3OT_17_ block terpolymer to form fibre-like micelles from the adsorbed seeds on gold substrates demonstrates that it is possible to grow near monodisperse fibre-like micelles by setting up an appropriate ratio between the number of adsorbed seeds and the concentration of unimer solution, and controlling the subsequent immersion time in the unimer solution. The length distributions of the fibre-like micelles can then be measured by AFM and understood on the basis of a chain-growth type of mechanism that is well-described by a Poisson process. This ability to control the length and chemical architecture of these structures from surface-adsorbed seeds represents an important feature of our approach, which complements the currently available bottom-up methods for assembling functional devices. Finally, a mesoscopic electrical device was demonstrated in which fibre-like micelles were grown across parallel microband electrodes and the current–voltage characteristics were switched between insulating and ohmic conduction by oxidative doping.

## Conflicts of interest

There are no conflicts to declare.

## Supplementary Material

Supplementary information
