# Supplementary material for: Bottom-up device fabrication via the seeded growth of polymer-based nanowires
Source: Chem Sci. 2020 Jun 1;11(24):6222–8. doi: 10.1039/d0sc02011g (PMC7480267; doi:10.1039/d0sc02011g)
Supplement: Supplementary file 1 [file SC-011-D0SC02011G-s001.pdf]

Electronic Supplementary Material (ESI) for Chemical Science.
This journal is © The Royal Society of Chemistry 2020

Bottom Up Device Fabrication via the Seeded Growth of Polymer-based Nanowires

Osama El-Zubir,[a] Emily L. Kynaston,[b] Jessica Gwyther,[b] Ali Nazemi,[b] Oliver E. C. Gould,[b] George R. Whittell,[b] Benjamin. R. Horrocks,[a] Ian Manners *[b, c] and Andrew Houlton *[a]

[a] *Chemical Nanoscience Labs, School of Natural and Environmental Sciences, Newcastle University, Newcastle upon Tyne NE1 7RU, UK.*

[b] *School of Chemistry, University of Bristol, Cantock's Close, Bristol BS8 1TS, UK.*

[c] *Department of Chemistry, University of Victoria, Victoria, V8W 3V6, British Columbia, Canada.*

** Corresponding author. Email: imanners@uvic.ca; andrew.houlton@ncl.ac.uk*

Supporting Information

Table of Content

MATERIALS AND METHODS **2**

- MATERIALS 2
- PROCEDURE FOR THE PREPARATION OF PFDMS$_{44}$-*B*-PDMS$_{250}$-*B*-P3OT$_{17}$ 2
- PREPARATION OF SEED MICELLES 3
- GROWTH OF FIBRE-LIKE MICELLES 4
- CONTROL OF SEED DENSITY AND FIBRE-LIKE MICELLE GROWTH 4
- ATOMIC FORCE MICROSCOPY (AFM) 4
- GOLD NANOGAP DEVICES 4
- ELECTRICAL MEASUREMENTS 5
- STATISTICAL ANALYSIS 5

CYCLIC VOLTAMMETRY STUDY TO ASSESS CHEMISORPTION OF SEEDS **5**

AFM STUDY TO ASSESS THE ATTACHMENT OF THE SEEDS TO THE GOLD SURFACE **6**

FIGURES FOR CONTROLLING THE GROWTH OF FIBRE-LIKE MICELLES **7**

GROWTH OF SELECTIVELY FUNCTIONALISED FIBRE-LIKE MICELLES ON GOLD SURFACES **9**

GROWTH OF FIBRE-LIKE MICELLES ON PLATINUM MBE FOR ELECTRICAL MEASUREMENTS **10**

CONTROL EXPERIMENT FOR DOPING THE FIBRE-LIKE MICELLES WITH NOBF$_4$ **11**

REFERENCES **11**

Materials and Methods

Materials

Gold coated silicon wafers (Substrata p-type <100>) were purchased from Substrata Thin Film Solutions. Acetonitrile (anhydrous, 99.8%, Sigma-Aldrich), decane (ReagentPlus®, ≥99%, Sigma-Aldrich), ethanol (absolute, HPLC, ≥99%, Fisher Scientific), lithium perchlorate (ACS reagent, ≥95.0%, Sigma-Aldrich), nitrosonium tetrafluoroborate (95%, Aldrich), and 2-Propanol (HPLC, ≥99.9%, Fisher Scientific). All chemicals were used as received unless otherwise specified.

Procedure for the preparation of PFDMS$_{44}$-*b*-PDMS$_{250}$-*b*-P3OT$_{17}$

The synthesis of PFDMS$_{44}$-b-PDMS$_{250}$-b-P3OT$_{17}$ is given as an example. Ethynyl-capped P3OT17 (29.0 mg, 0.008 mmol, 3300 g mol^{-1}, 1.1 eq.)[1] and azide-terminated PFDMS$_{44}$-b-PDMS$_{250}$ (200 mg, 0.007 mmol, 29,200 g mol^{-1}, 1 eq.)[1] were dissolved in dry THF (5 ml) in a 50 ml greaseless Schlenk flask equipped with a magnetic stirrer bar. PMDETA (20 µl, 0.0001 mmol) was added and the solution was freeze-pump-thawed in liquid N_2 for 3 cycles, allowed to warm to room temperature and transferred to an Argon-filled glovebox. CuBr (ca. 10 mg) was added and the flask was transferred back to the Schlenk line. The reaction was allowed to proceed at 50 °C for 48 h after which the solution was filtered through basic alumina. The crude product was isolated by precipitation into MeOH (3 times). The PFDMS$_{44}$-b-PDMS$_{250}$-b-P3OT$_{17}$ triblock terpolymer was isolated as a dark-red, gummy solid after SEC purification. Yield = 65.0 mg, 28 %. ^{1}H-NMR integration together with the absolute molar mass of the PFDMS block (number-averaged molecular weight (Mn) = 10,600 and Mw/Mn = 1.09) afforded the block ratio and various degrees of polymerisation (see Fig. S1). Mw/Mn was shown to be 1.09 (size exclusion chromatography (SEC), Fig. S2).

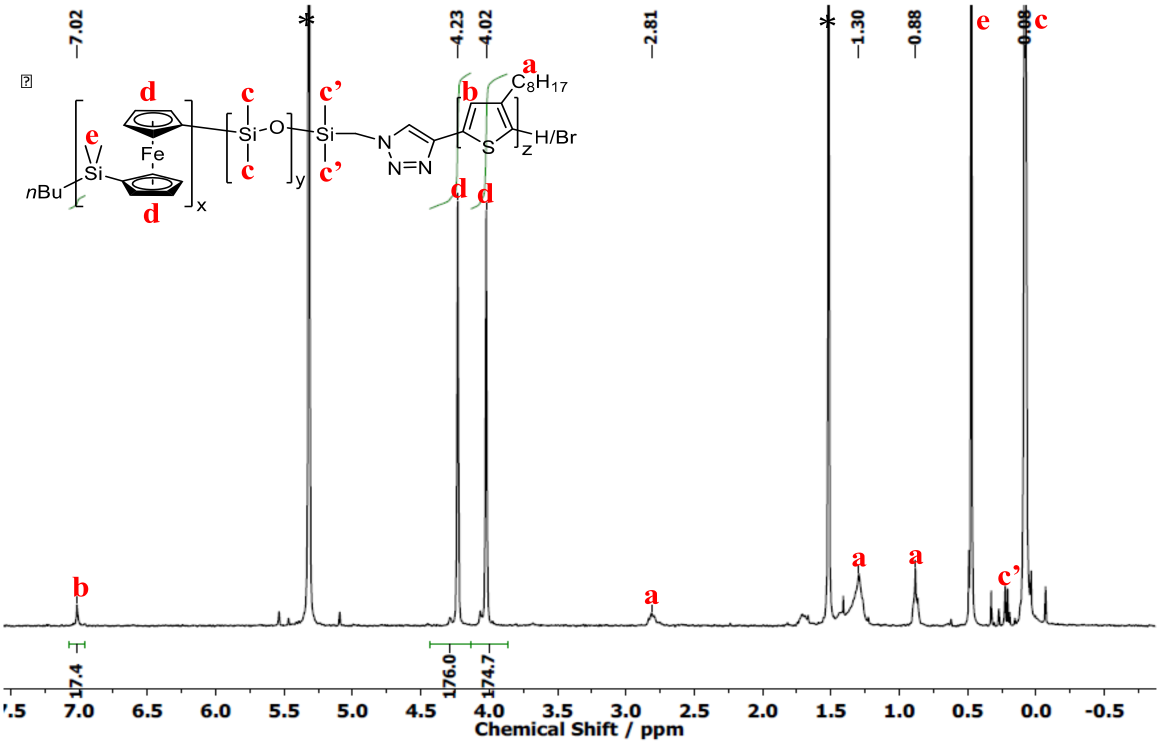

Fig. S1. ^{1}H-NMR (400 MHz, CD_2Cl_2) spectrum of PFDMS$_{44}$-*b*-PDMS$_{250}$-P3OT$_{17}$. * denotes residual solvents (CH_2Cl_2 and H_2O).

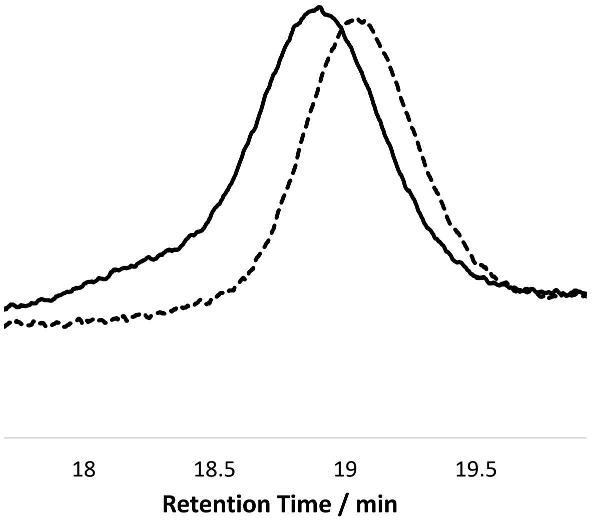

Fig. S2. SEC trace of $PFDMS_{44}$-b-$PDMS_{250}$-N_3 and $PFDMS_{44}$-b-$PDMS_{250}$-$P3OT_{17}$. SEC trace (RI response) of $PFDMS_{44}$-b-$PDMS_{250}$-N_3 (dashed line) and $PFDMS_{44}$-b-$PDMS_{250}$-$P3OT_{17}$ (solid line) measured in THF at 35 °C.

Preparation of seed micelles

$PFDMS_{44}$-*b*-$PDMS_{250}$-*b*-$P3OT_{17}$ was suspended in decane (0.25 mg ml^{-1}) in a 7 ml vial sealed with a screw-cap. The suspension was heated to 120 °C for 1 h in a block heater and allowed to cool slowly to room temperature (23 °C). Samples were aged for 24 h before aliquots were drop-cast onto carbon-coated copper-grids for TEM analysis.

A solution of $PFDMS_{44}$-*b*-$PDMS_{250}$-*b*-$P3OT_{17}$ micelles in decane (0.25 mg ml^{-1}) was subjected to ultrasonication for 3 h (6 x 30 mins) in a sonic cleaning bath, taking care to not let the micelle solution overheat (sonication temperature range 20 – 30 °C). An aliquot was taken and prepared for TEM analysis. Micelle measurements taken from the TEM images and subsequent statistical analysis indicated the seed micelles were 50 nm (L_w/L_n = 1.16). Longer $PFDMS_{44}$-*b*-$PDMS_{250}$-*b*-$P3OT_{17}$ seed micelles were prepared by adding 4 equivalents (m_{unimer}/m_{seed} = 4) of a solution of $PFDMS_{44}$-*b*-$PDMS_{250}$-b-$P3OT_{17}$ in THF (5 mg ml^{-1}) to the sonicated seeds. Statistical analysis on TEM images of this sample suggested the elongated seed micelles were 300 nm (L_w/L_n = 1.05).

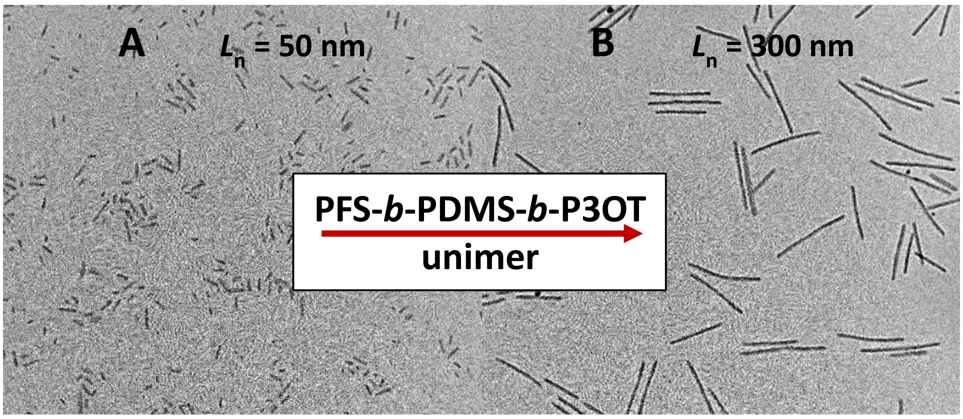

Fig. S3. TEM images of short and longer $PFDMS_{44}$-b-$PDMS_{250}$-b-$P3OT_{17}$ seed micelles. Representative bright-Field TEM images of (A) $PFDMS_{44}$-b-$PDMS_{250}$-b-$P3OT_{17}$ seed micelles drop-cast from decane (50 nm, 0.25 mg ml^{-1}). (B) Longer $PFDMS_{44}$-b-$PDMS_{250}$-b-$P3OT_{17}$ seed micelles drop-cast from decane (300 nm, 0.25 mg ml^{-1}). Each image is approximately 2 µm x 2 µm.

Growth of fibre-like micelles

A gold coated silicon wafer (Substrata p-type <100>) was fabricated by depositing a 50 nm layer of 99.999% pure gold onto a layer of 5 nm chromium (99.99%) as an adhesion promoter, using electron beam evaporation. The gold coated silicon wafer was cut to 8 x 8 mm^2 pieces and washed with ethanol. The gold-coated substrates were sonicated in ethanol for 15 min, dried with nitrogen gas and then exposed to oxygen plasma (Femto - diener electronic, Plasma-Surface-Technology) for 10 min at 80 W. After this the substrates were immersed in a decane solution of fibre-like micelle seeds. The concentration and immersion period to achieve the desired number of seeds per micrometer was adjusted as required. After immersion in the seed solution the substrates were rinsed with decane and immersed in a decane solution of unimer. Again the concentration and immersion time was varied to optimise the growth of the fibre-like micelles to the desired length. The samples were rinsed again with decane and dried with nitrogen gas before characterisation. It was noted that seeds can be kept active in THF at room temperature for up to 6 months.

Control of seed density and fibre-like micelle growth

The gold-coated substrates were washed and sonicated, dried and exposed to an oxygen plasma. In the control of seed density experiment, the substrates were immersed for 2 h in 0.02, 0.1, 0.2, 0.4 and 1 µg ml^{-1} decane solutions of micelle seeds. In the experiment to control the micelle length by variation of unimer concentration, seeds were adsorbed onto the substrates by immersion for 2 h in 0.02 µg ml^{-1} solutions. These substrates were then washed with decane and immersed for 18 h in 0.5, 1, 2 and 4 µg ml^{-1} unimer solutions. In the experiment to monitor the micelle length by variation of immersion time of adsorbed seeds in unimer solution, seeds were adsorbed by immersion for 2 h in a 0.005 µg ml^{-1} seeds solution. The adsorbed seeds were left to grow for 2, 4, 6, 8, 10 days in 0.5 µg ml^{-1} decane solutions of unimer. All samples were washed with decane and dried in a stream of nitrogen gas before characterisation.

Atomic Force Microscopy (AFM)

A Digital Instruments Multimode-8 with Nanoscope V controller and E scanners (Bruker) was used for acquiring AFM images. Nanoscope software version 9.1 was used to control the microscope. The AFM data were analysed with NanoScope Analysis 1.50 software (Bruker). The AFM was operated in ScanAsyst in air and tapping modes. An isolation table (Veeco Inc., Metrology Group) was used to minimise vibrational noise. Silicon tips on silicon nitride cantilevers (ScanAsyst-Air, Bruker) were used for imaging in ScanAsyst mode. The nominal tip curvature radius was approximately 2 nm, resonant frequency ~70 kHz and spring constant k ~0.4 Nm^{-1}. For imaging in taping mode, silicon tips on silicon cantilevers (Tap300Al-G, BudgetSensors) were used for imaging. The nominal tip curvature radius was approximately <10 nm, resonant frequency ~300 kHz and spring constant k ~40 Nm^{-1}.

Gold nanogap devices

Gold nanogaps were fabricated in the laboratory of Prof Douglas Paul, School of Engineering, University of Glasgow. A 200 nm SiO_2 layer was grown on 20 x 20 mm Si substrate. The SiO_2 layer was Spin coated with a bilayer of polymethylmethacrylate PMMA. A Vistec VB6 electron beam lithography tool was used to pattern the PMMA bilayer and then the pattern was developed. A 10 nm titanium and 100 nm gold were deposited over the patterned PMMA layer. The PMMA layer was lifted-off, leaving 2 mm squares of gold film with a 600 nm gap. The Au nanogaps were washed with ethanol and dried with nitrogen gas. They were then exposed to oxygen plasma for 10 min at 80 W. After exposure to oxygen plasma, the electrodes were immersed immediately in 0.005 µg ml^{-1} solution of seeds in decane for 1 h. After rinsing with decane, they were immersed in 1 µl ml^{-1} solution of unimer in decane for 5 days. The devices were rinsed again with decane and dried with nitrogen gas.

Electrical measurements

Current-voltage measurements were carried out on a probe station (Cascade Microtech with a B1500A parameter analyser, Agilent). All the electrical data were collected under dry nitrogen without light illumination. For the electrical measurements, the micelles were grown from adsorbed seeds on platinum microband electrodes (MBEs; Smart Microsystems Pt MB-4000, Windsor Scientific Ltd. Slough, UK). Four parallel platinum electrodes were fabricated on Si/SiO_2 substrates. The width of electrodes is 10 µm with 10 µm gaps between them and the height is 200 nm above the intervening oxide. The surfaces of the MBEs were electrically insulated except for a 2 x 2 mm^2 area for growing the micelles and the area for the contact pads for connection to the external circuit. The platinum MBEs were washed with ethanol and dried with nitrogen gas. The clean platinum electrodes were investigated on a probe station and reference I-V curves were collected that showed the background currents to be less than 100 fA at 2 V. Nanowired devices were fabricated by immersion of the clean platinum MBEs in a 0.1 µg ml^{-1} solution of seeds in decane for 10 min. After adsorption of seeds, the electrodes were washed with decane and immersed in 10 µl ml^{-1} solution of unimers in decane for 7 days to grow a sufficient number of micelles across the gap between the electrodes. The device was washed again with decane and dried with nitrogen gas. The device was connected to the probe station and current/voltage curves were collected. For doping, the micelles on MBEs were treated with a drop (~1 µl) of 0.1 mol l^{-1} of nitrosonium tetrafluoroborate ($NOBF_4$) in isopropanol. The $NOBF_4$ drop was left for 20 min and dried with nitrogen gas before the current/voltage curves were collected. $NOBF_4$ is was chosen as the oxidant because the reduction product (NO) is gaseous and rapidly removed from the surface.

Statistical analysis

The AFM data were analysed with NanoScope Analysis 1.50 software (Bruker). All data were determined from at least 6 AFM images on randomly selected areas of each sample.

Cyclic voltammetry study to assess chemisorption of seeds

Cyclic voltammetry (CV) was performed on gold electrodes after immersion in a solution of $PFDMS_{44}$-*b*-$PDMS_{250}$-*b*-$P3OT_{17}$ micelles or seeds in decane (0.1 mg/mL) for 1 h followed by washing with iPrOH. The CVs were recorded using a standard three-electrode cell in an electrolyte of LiClO4 (0.1 mol l^{-1}) in acetonitrile. The potentiostat was an EmStat 3 (PalmSens) and the electrodes were a platinum wire counter electrode, a 2 mm diameter gold disc working electrode, and a non-aqueous Ag/0.01M Ag^+ (acetonitrile) reference electrode.

In order to assess the chemisorption of $PFDMS_{44}$-*b*-$PDMS_{250}$-*b*-$P3OT_{17}$ seeds on gold surfaces, we used cyclic voltammetry (CV). Fig. S4A shows the CV measurement of $PFDMS_{44}$-*b*-$PDMS_{250}$-*b*-$P3OT_{17}$ seeds that was adsorbed on a gold electrode by immersion in a $PFDMS_{44}$-*b*-$PDMS_{250}$-*b*-$P3OT_{17}$ seeds solution in decane (0.1 mg/ml) for 1 h and then the electrode washed with iPrOH. The CV data shows two anodic peaks which attributed to ferrocene core-block oxidation at 0.15 V and 0.34 V. The two reversible one-electron oxidation waves may be due to the interaction between the iron atoms in the polyferrocene.[2] In fig. S4B, the CV measurements were recorded for the seeds on the gold electrode before (blue) and after addition of 50 nmol l^{-1} ferrocene solution (red) and 500 nmol l^{-1} ferrocene solution (green) to the LiClO4 solution (0.1 mol l^{-1}) in acetonitrile, as a control experimental. After the addition of 50 nmol l^{-1} ferrocene, there was no change in the positions of oxidation peaks. However, the base of the first oxidation beak at 0.15 V became wider because of the single ferrocene in the solution. The first oxidation peak is sharper than the second peak may be because thiophene–related component overlapped with ferrocene first oxidation peak. The thiophene oxidation peak is expected to be at about 0.22 V, since it was reported in literature that E_{ox} of P3OT is 0.72 V versus Ag/AgCl reference electrode.[3, 4] In this study, the potential was measured versus a non-aqueous Ag/Ag^+ electrode as a reference electrode. It has a potential of 0.68 V vs SHE in water. The difference between

the two reference electrodes is about 0.44 V. After the ferrocene concentration was increased to 500 nmol l^{-1}, the wide peak of the ferrocene in the solution has covered the polyferrocene peaks.

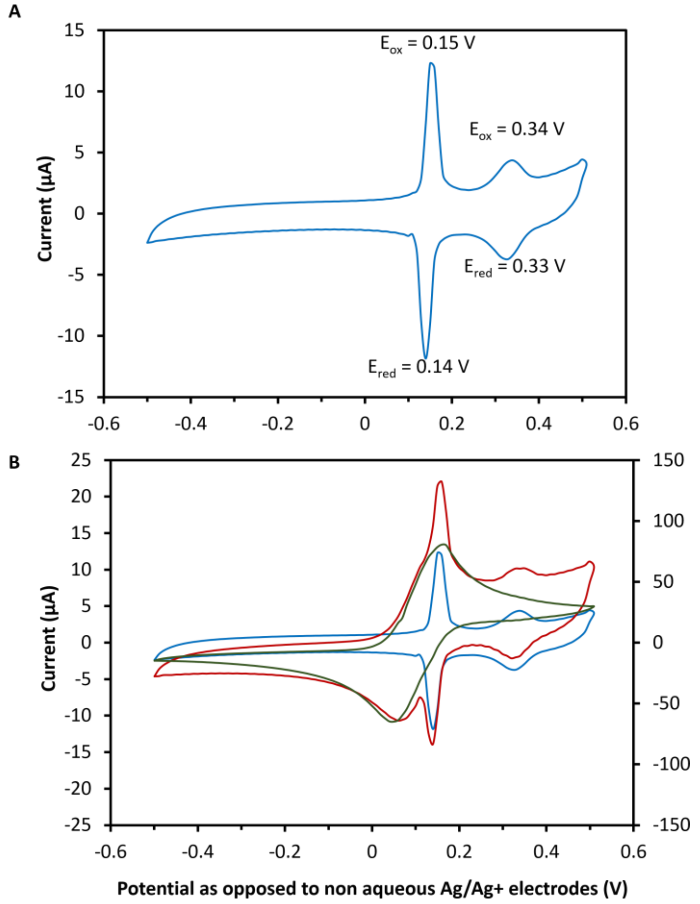

Fig. S4. CV measurements for PFDMS$_{44}$-b-PDMS$_{250}$-b-P3OT$_{17}$. (A) Seeds and (B) seeds (blue) and seeds and different concentration of ferrocene (red is seeds + 50 nmol ml^{-1} ferrocene and green is seeds + 500 nmol ml^{-1} ferrocene (secondary axis)). CV measurements were measured using a standard three-electrode cell in acetonitrile with 0.1 mol l^{-1} $LiClO_4$, platinum counter electrode, gold electrode, and non-aqueous Ag/Ag^+ reference electrode. The Au electrode was immersed in a solution of PFDMS$_{44}$-b-PDMS$_{250}$-b-P3OT$_{17}$ seeds in decane (0.1 mg/ml) for 1 h and washed with iPrOH before the CV measurement.

AFM study to assess the attachment of the seeds to the gold surface

In order to confirm that the micelles were self-assembled from immobilised PFDMS$_{44}$-*b*-PDMS$_{250}$-*b*-P3OT$_{17}$ seeds on surface, not from floated seeds in unimer solution and then reattach to the gold surface on drying, the attachment of the seeds to the gold surface was examined by AFM after immersion in pure decane for different period time. A gold coated silicon slide (8 x 8 mm^2) was marked with cross in the centre to find the same site under the AFM camera. The sample was sonicated in ethanol, dried with nitrogen gas and exposed to oxygen plasma. Then, the sample was immersed for 2 h in a decane solution of PFDMS$_{44}$-*b*-PDMS$_{250}$-*b*-P3OT$_{17}$ seeds (0.5 µg ml^{-1}). After adsorption of the seeds, the surface was washed by immersion in pure decane for 2 min and dried with nitrogen gas. The immobilised seeds on gold surface was scanned and a site with a landmark (aggregation of seeds at the top right of the image in Fig. S5A-E) was located using the AFM. After the first scan, the surface was immersed again in pure decane (the total immersion time was 2, 4, 6 and 24 h) and the same site was scanned after each immersion. The AFM data in Fig. S5A-E confirm that the PFDMS$_{44}$-*b*-PDMS$_{250}$-*b*-P3OT$_{17}$ seeds are strongly attached to the gold surface, are not desorbed or mobile in decane and the seeded self-assembly of micelles therefore takes place on the gold surface. Fig. S5F shows a control sample of a pre-cleaned gold substrate immersed in a decane solution (2 µg ml^{-1}) of unimers for 18 h.

The AFM image shows no micelles grown on the surface. That confirms the living-CDSA process by self-initiation is inefficient compared to surface-seeded growth of micelles over this timeframe.

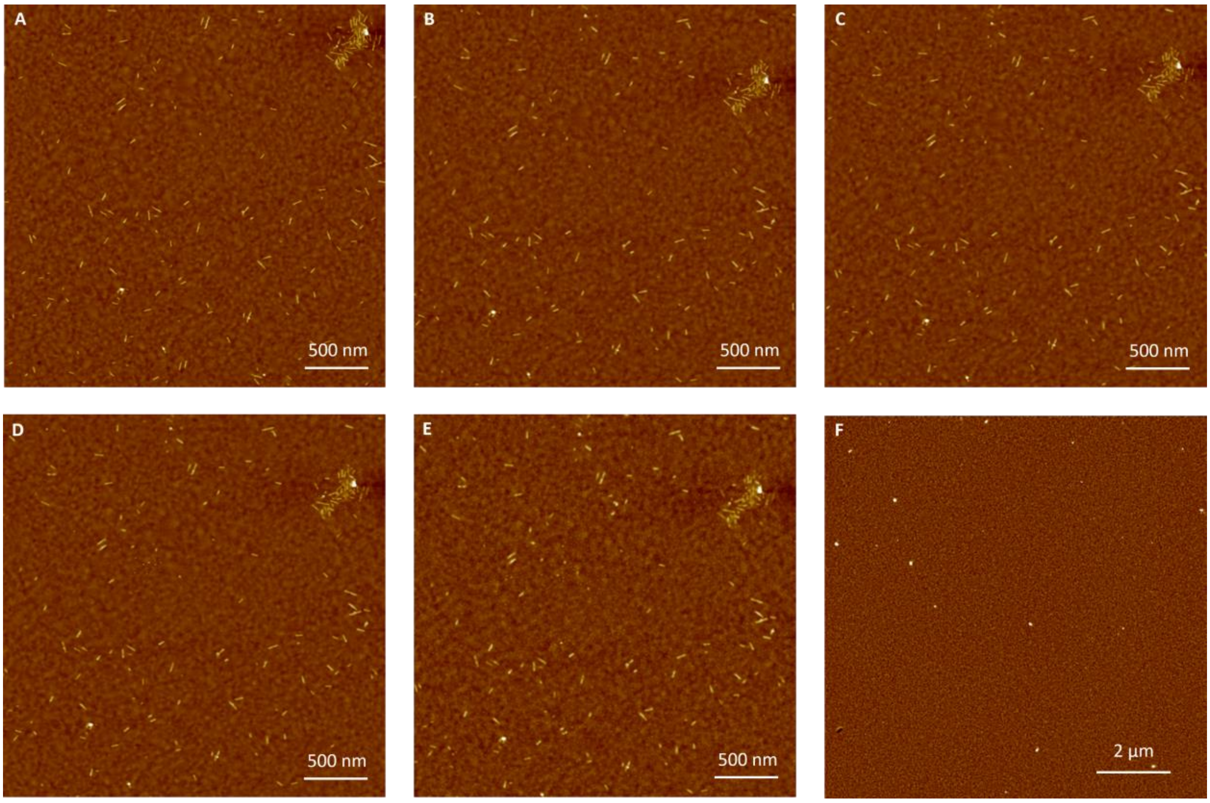

Fig. S5. AFM images of immobilised PFDMS$_{44}$-*b*-PDMS$_{250}$-*b*-P3OT$_{17}$ seeds on gold surface and a control sample. The seeds were adsorbed on gold surface from 0.5 µg ml^{-1} solution of PFDMS$_{44}$-*b*-PDMS$_{250}$-*b*-P3OT$_{17}$ seeds in decane. The immobilised seeds on gold coated silicon slides were immersed for (A) 2 min, (B) 2 h, (C) 4h, (D) 6 h and (E) 24h in pure decane. (F) AFM image of a control sample prepared by immersion of the bare gold substrate in 2 µg ml^{-1} solution of unimers (no seeds) for 18 h.

Figures for controlling the growth of fibre-like micelles

Here we include the following figures showing the controlled growth of micelles from different concentration of unimers solution (Fig. S6) and slow growth from lower concentration of seeds solutions (Fig. S7).

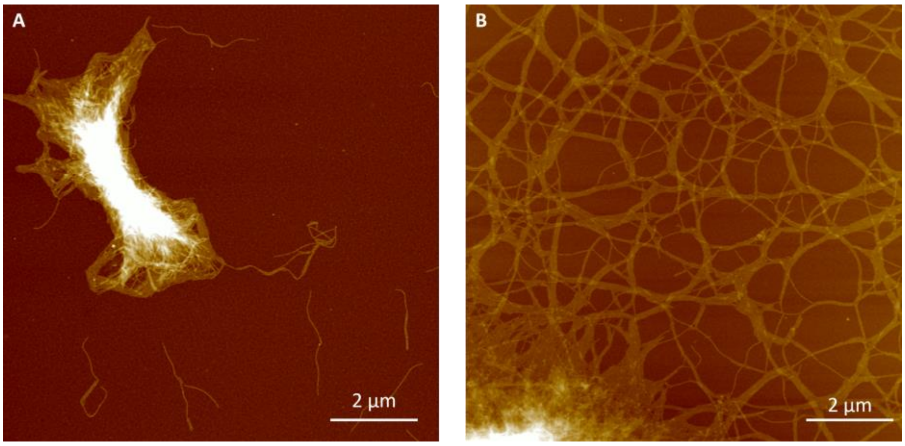

Fig. S6. AFM images of fibre-like micelles were grown from concentrated unimer solutions. The micelles were grown up from 0.02 µg ml^{-1} immobilised seeds of PFDMS$_{44}$-*b*-PDMS$_{250}$-*b*-P3OT$_{17}$ on gold surfaces by immersion in different concentration of unimers solution of PFDMS$_{44}$-*b*-PDMS$_{250}$-*b*-P3OT$_{17}$ (A) 4 µg ml^{-1} and (B) 6 µg ml^{-1} for overnight.

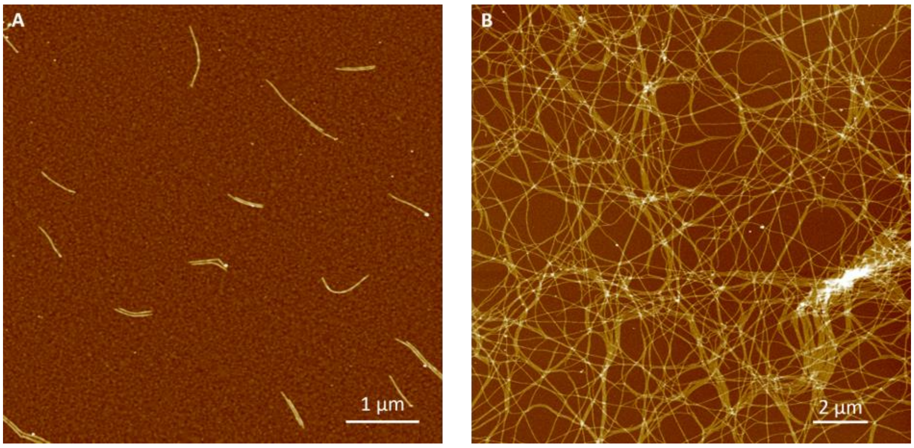

Fig. S7. AFM images of fibre-like micelles were grown up slowly from lower concentration. The micelles have been grown up from 20 ng/ml immobilised seeds on gold by immersion in 2 µg/ml solution of unimers for (A) a day and (B) 20 days.

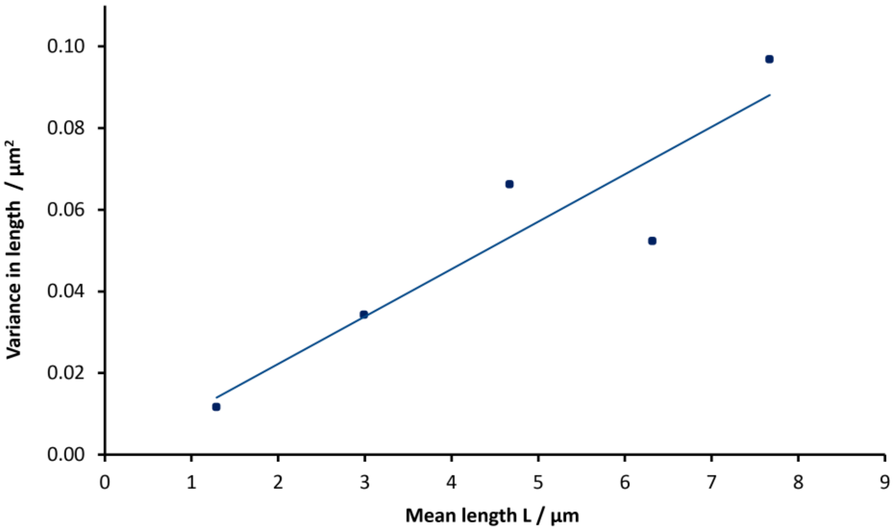

Fig. S8. Analysis of the variance and mean of the length distributions for surface-grown fibre-like micelles. The micelles were seeded on gold surfaces from a 5 ng/ml seed solution and grown by immersion in 0.5 µg/ml unimer solution. Data from Fig. 2I-L.

Growth of selectively functionalised fibre-like micelles on gold surfaces

Fig. S9 summarises the growth of diverse functionalised micelles from adsorbed PFDMS$_{44}$-*b*-PDMS$_{250}$-*b*-P3OT$_{17}$ seeds by adding different unimers. The micelles in Fig. S9A were formed from adsorbed, P3OT functionalised seeds that were immersed in 4 µg ml^{-1} solution of PFDMS$_{44}$-*b*-PDMS$_{250}$-*b*-P3NSe$_{17}$ unimers for overnight. These micelles were self-assembled to about 2.8 µm in length. The micelles in Fig. S9B were self-assembled from adsorbed triblock terpolymer (PFDMS$_{44}$-*b*-PDMS$_{250}$-*b*-P3OT$_{17}$) seeds (0.005 µg ml^{-1}) that were immersed in 2 µg ml^{-1} solution of diblock terpolymer (PFDMS$_{44}$-*b*-PDMS$_{250}$-*b*-N$_3$) for overnight. Diblock terpolymer micelles functionalised with azide groups were self-assembled on the gold surface to about 2.4 µm in length. The diblock terpolymer micelles (Fig. S9B) were immersed again after the AFM scan in 1 µg ml^{-1} solution of triblock terpolymer (PFDMS$_{44}$-*b*-PDMS$_{250}$-*b*-P3OT$_{17}$) in decane for overnight. These micelles (Fig. S9C) were grown to about 5.3 µm in length. As in solution[5, 6], selective regions of growing micelle can be selectively functionalised with different groups by growth of the poly(ferrocenyldimethylsilane) core using different di or triblock terpolymer with active ends.

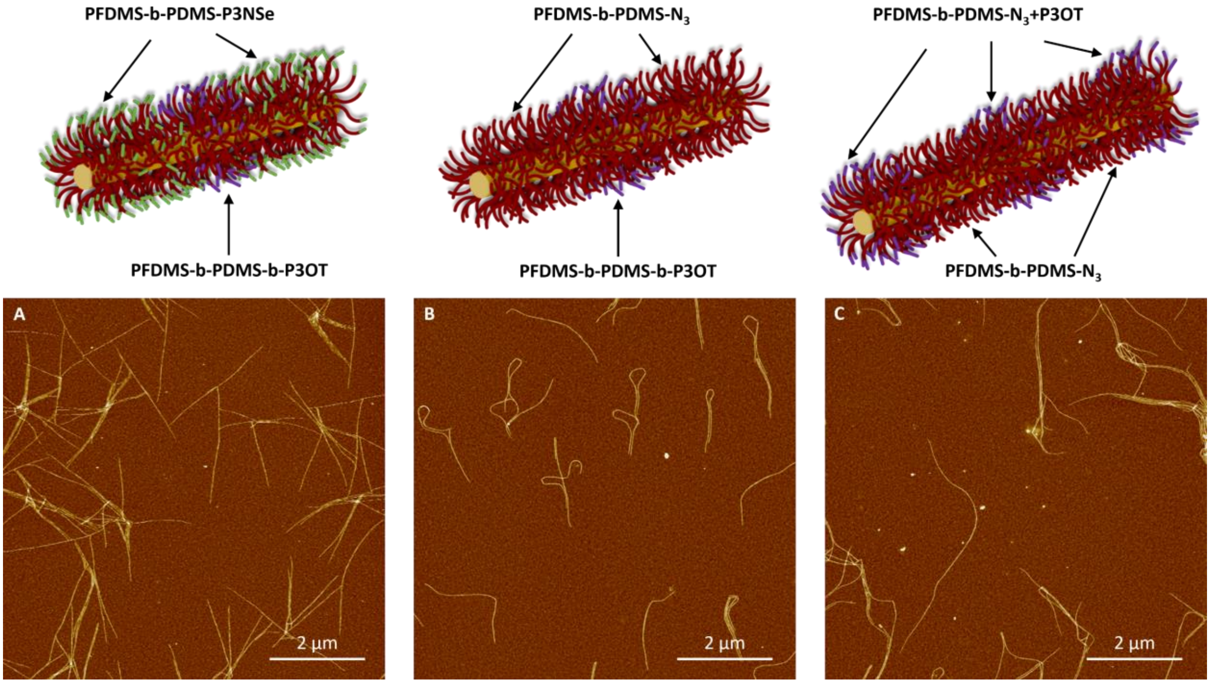

Fig. S9. AFM images of fibre-like micelles that have been grown on gold surfaces from different unimer solutions. The micelles were grown from adsorbed seeds of PFDMS$_{44}$-*b*-PDMS$_{250}$-*b*-P3OT$_{17}$ on gold surfaces by immersion in different solutions of unimers: (a) 4 µg ml^{-1} of PFDMS$_{44}$-*b*-PDMS$_{250}$-*b*-P3NSe $_{17}$, (b) 2 µg ml^{-1} of PFDMS$_{44}$-*b*-PDMS$_{250}$-N$_3$ and (c) 2 µg ml^{-1} of PFDMS$_{44}$-*b*-PDMS$_{250}$-N$_3$ and after that 1 µg ml^{-1} of PFDMS$_{44}$-*b*-PDMS$_{250}$-*b*-P3OT$_{17}$ for overnight.

Growth of fibre-like micelles on platinum MBE for electrical measurements

For electrical measurements, terpolymer micelles were grown from adsorbed seeds on parallel platinum microband electrodes (MBE) with a 10 µm inter-electrode gap. The platinum MBE was immersed in a 0.1 µg ml^{-1} seed solution in decane for 10 min followed by immersion for 7 days in a 10 µg ml^{-1} unimer solution in decane. This allowed a sufficient number of bundles of fibre-like micelles to grow across the inter-electrode gaps for subsequent electrical measurements.

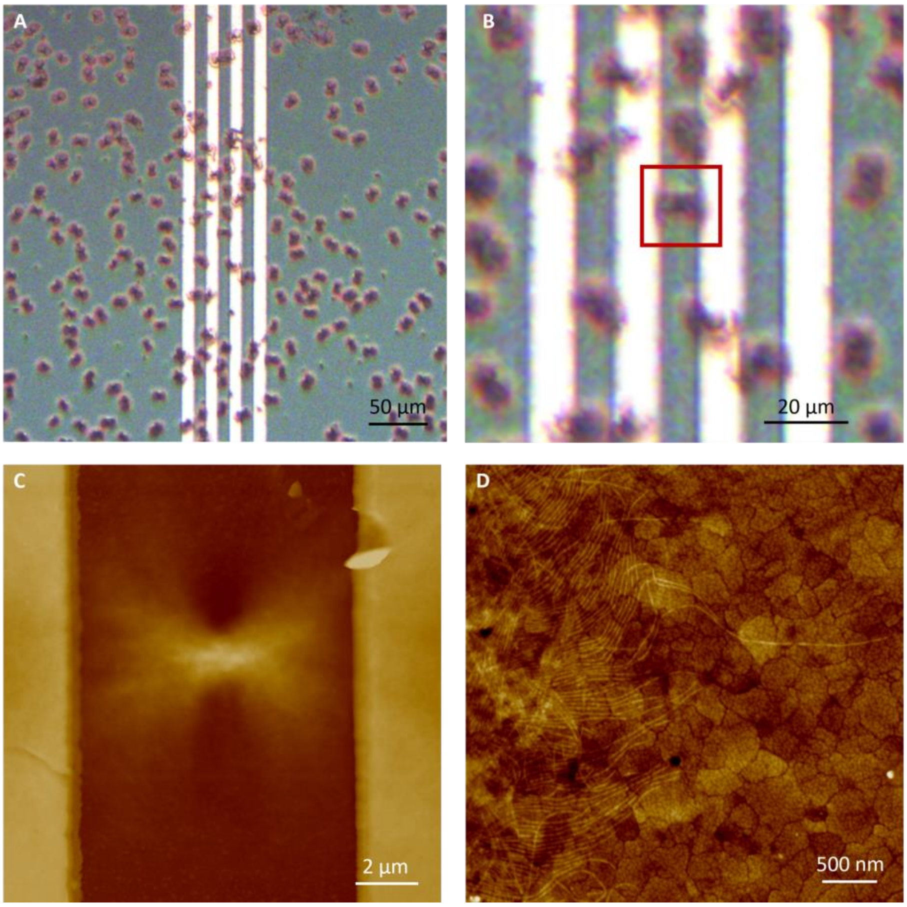

Fig. S10. Growth of micelles on Pt microband electrodes. Optical microscope image (A) and a zoom area (B) of bundles of fibre-like micelles grown from adsorbed seeds on platinum MBE. (C) AFM topographical image of a bundle of fibre-like micelles crossing the inter-electrode 10 µm gap (red box in image B). (D) Higher resolution AFM image showing micelles on the Pt electrode.

Control experiment for doping the fibre-like micelles with $NOBF_4$

control experiment was carried out to test for the contribution of residual oxidant to the conductance. We applied $NOBF_4$ to MBEs in the absence of micelles. The I-V measurements for both clean and control MBEs showed a non-conductive behaviour and background currents in the fA range.

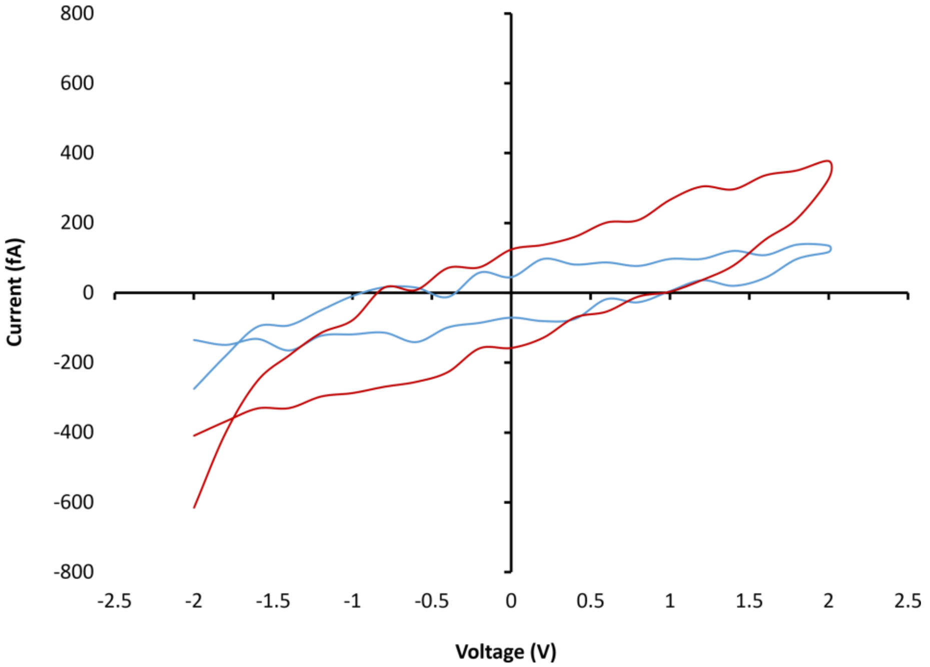

Fig. S11. I-V Measurements of the $NOBF_4$ control. The I-V measurement was recorded on clean platinum microband electrodes (blue) and after $NOFB_4$ was applied to the microband electrodes (red).

References

1 U. Tritschler, J. Gwyther, R. L. Harniman, G. R. Whittell, M. A. Winnik and I. Manners, *Macromolecules*, 2018, **51**, 5101-5113.

2 R. Petersen, D. A. Foucher, B. Z. Tang, A. Lough, N. P. Raju, J. E. Greedan and I. Manners, *Chem. Mater.*, 1995, **7**, 2045-2053.

3 D. C. Bento, E. C. R. Maia, T. N. M. Cervantes, R. V. Fernandes, E. Di Mauro, E. Laureto, M. A. T. da Silva, J. L. Duarte, I. F. L. Dias and H. de Santana, *Synth. Met.* , 2012, **162**, 2433-2442.

4 T. N. M. Cervantes, D. C. Bento, E. C. R. Maia, R. V. Fernandes, E. Laureto, G. J. Moore, G. Louarn and H. de Santana, *J. Mater. Sci.: Mater. Electron.*, 2014, **25**, 1703-1715.

5 H. Wang, W. J. Lin, K. P. Fritz, G. D. Scholes, M. A. Winnik and I. Manners, *J Am Chem Soc*, 2007, **129**, 12924-12925.

6 X. S. Wang, G. Guerin, H. Wang, Y. S. Wang, I. Manners and M. A. Winnik, *Science*, 2007, **317**, 644-647.
